# Does the *KIR2DS5* Gene Protect from Some Human Diseases?

**DOI:** 10.1371/journal.pone.0012381

**Published:** 2010-08-26

**Authors:** Izabela Nowak, Edyta Majorczyk, Andrzej Wiśniewski, Andrzej Pawlik, Maria Magott-Procelewska, Ewa Passowicz-Muszyńska, Jacek Malejczyk, Rafał Płoski, Sebastian Giebel, Ewa Barcz, Aleksandra Zoń-Giebel, Andrzej Malinowski, Henryk Tchórzewski, Arkadiusz Chlebicki, Wioleta Łuszczek, Maciej Kurpisz, Marian Gryboś, Jacek Wilczyński, Piotr Wiland, David Senitzer, Ji-Yao Sun, Renata Jankowska, Marian Klinger, Piotr Kuśnierczyk

**Affiliations:** 1 Department of Clinical Immunology, Institute of Immunology and Experimental Therapy, Polish Academy of Sciences, Wrocław, Poland; 2 Department of Pharmacokinetics and Therapeutic Drug Monitoring, Pomeranian Medical University, Szczecin, Poland; 3 Department and Clinic of Nephrology and Transplantation Medicine, Faculty of Medicine, Wrocław Medical University, Wrocław, Poland; 4 Department of Pulmonology and Lung Cancer, Wrocław Medical University, Wrocław, Poland; 5 Department of Histology and Embryology, Centre of Biostructure Research, Medical University of Warsaw, Warszawa, Poland; 6 Department of Medical Genetics, Centre of Biostructure Research, Warsaw Medical University, Warszawa, Poland; 7 Department of Clinical and Experimental Oncology, Comprehensive Cancer Centre, Maria Sklodowska-Curie Memorial Institute Branch Gliwice, Gliwice, Poland; 8 Department of Hematology and Bone Marrow Transplantation, Silesian Medical University, Katowice, Poland; 9 1st Chair and Clinic of Obstetrics and Gynecology, Medical University of Warsaw, Warszawa, Poland; 10 Silesian Hospital of Rheumatology and Rehabilitation, Ustroń, Poland; 11 Department of Surgical and Endoscopic Gynecology, Polish Mother's Memorial Hospital-Research Institute, Łódź, Poland; 12 Department of Clinical Immunology, Polish Mother's Memorial Hospital-Research Institute, Łódź, Poland; 13 Chair and Clinic of Rheumatology and Internal Diseases, Wrocław Medical University, Wrocław, Poland; 14 Department of Reproductive Biology and Stem Cells, Institute of Human Genetics, Polish Academy of Sciences, Poznań, Poland; 15 1st Department of Gynecology and Obstetrics, Medical University of Wrocław, Wrocław, Poland; 16 Department of Gynecological Surgery, Polish Mother's Memorial Hospital-Research Institute, Łódź, Poland; 17 City of Hope Comprehensive Cancer Center, Duarte, California, United States of America; 18 Jan Długosz Pedagogical University, Częstochowa, Poland; Centre de Recherche Public de la Santé, Luxembourg

## Abstract

**Background:**

*KIR2DS5* gene encodes an activating natural killer cell receptor whose ligand is not known. It was recently reported to affect the outcome of hematopoietic stem cell transplantation.

**Methodology/Principal Findings:**

In our studies on *KIR2DS5* gene associations with human diseases, we compared the frequencies of this gene in patients and relevant controls. Typing for *KIR2DS5* gene was performed by either individual or multiplex polymerase chain reactions which, when compared in the same samples, gave concordant results. We noted an apparently protective effect of *KIR2DS5* gene presence in several clinical conditions, but not in others. Namely, this effect was observed in ankylosing spondylitis (*p* = 0.003, odds ratio [OR] = 0.47, confidence interval [CI] = 0.28–0.79), endometriosis (*p* = 0.03, OR = 0.25, CI = 0.07–0.82) and acute rejection of kidney graft (*p* = 0.0056, OR = 0.44, CI = 0.24–0.80), but not in non-small-cell lung carcinoma, rheumatoid arthritis, spontaneous abortion, or leukemia (all *p*>0.05). In addition, the simultaneous presence of *KIR2DS5* gene and *HLA-C* C1 allotype exhibited an even stronger protective effect on ankylosing spondylitis (*p* = 0.0003, OR = 0.35, CI = 0.19–0.65), whereas a lack of *KIR2DS5* and the presence of the *HLA-C* C2 allotype was associated with ankylosing spondylitis (*p* = 0.0017, OR = 1.92, CI = 1.28–2.89), whereas a lack of KIR2DS5 and presence of C1 allotype was associated with rheumatoid arthritis (*p* = 0.005, OR = 1.47, CI = 1.13–1.92). The presence of both *KIR2DS5* and C1 seemed to protect from acute kidney graft rejection (*p* = 0.017, OR = 0.47, CI = 0.25–0.89), whereas lack of *KIR2DS5* and presence of C2 seemed to favor rejection (*p* = 0.0015, OR = 2.13, CI = 1.34–3.37).

**Conclusions/Significance:**

Our results suggest that KIR2DS5 may protect from endometriosis, ankylosing spondylitis, and acute rejection of kidney graft.

## Introduction

Killer immunoglobulin-like two-domain short-tail receptor 5 (KIR2DS5) is a member of a large KIR family of cell-surface receptors expressed on natural killer (NK) cells and subpopulations of T lymphocytes. In contrast to long cytoplasmic tail-possessing KIRs (KIR2DL and KIR3DL), which inhibit cell activation upon ligand binding, KIRs with short cytoplasmic tails (KIR3DS1 and KIR2DS, including KIR2DS5) activate cells expressing them. Ligands (HLA class I molecules) are known only for some KIRs (HLA-C alleles from the C1 group [Asn80] for KIR2DL2 and KIR2DL3; HLA-C alleles from the C2 group [Lys80] for KIR2DL1 and KIR2DS1; HLA-B from the Bw4 group for KIR3DL1 and possibly KIR3DS1; HLA-A*03, *11 for KIR3DL2), whereas no physiological ligands are known for the remaining KIRs, including KIR2DS5 [1], [2]. Nevertheless, the effects of *KIR2DS5* gene presence or absence in the context of HLA-C C1 or C2 or both were recently observed in bone marrow transplantation [3]. Upon our studies on the associations of KIR genes with a panel of autoimmune, gynecological, and neoplastic diseases as well as in the acute rejection of kidney graft, we observed effects of *KIR2DS5* gene and *HLA-C* C1 and C2 groups in some of these clinical situations. A protective effect of *KIR2DS5* gene on susceptibility to psoriasis vulgaris was already described elsewhere [4]. Other results are presented here.

## Results

The frequencies of *KIR2DS5* gene in autoimmune and neoplastic diseases as well as in renal transplant rejection were compared with its frequency in a representative population of 690 healthy unrelated Poles (Control I, see Materials and Methods). Significant differences were found in only two clinical situations, ankylosing spondylitis (AS) and acute rejection of kidney graft (Table 1). In both instances a decrease in *KIR2DS5* frequency was observed. However, only AS result was still significant after Bonferroni correction (p_c_ = 0.033). No significance was found in comparisons between controls (Control I) and patients with rheumatoid arthritis (RA), stable kidney graft function, and neoplastic diseases such as non-small-cell lung carcinoma (NSCLC), acute lymphoblastic leukemia (ALL), acute myeloid leukemia (AML), and chronic myeloid leukemia (CML).

**Table 1:** Frequencies of *KIR2DS5* gene in different diseases.

Group	N	% (positive)	*p* value	Odds ratio	Confidence intervals	Statistical power (%)****
Control I*	690	29.7 (205)				
Control II**	213	31.9 (68)				
Control III***	117	31.6 (37)				
**Patients group vs Control I**						
Ankylosing spondylitis	115	16.5 (19)	0.003	0.47	0.28–0.79	87
Rheumatoid arthritis	366	24.3 (89)	0.07	0.76	0.57–1.02	46
Non-small cell lung carcinoma	280	25.7 (72)	>0.05	-	-	24
Acute lymphoblastic leukemia	22	23.8 (5)	>0.05	-	-	13
Acute myeloid leukemia	40	32.5 (13)	>0.05	-	-	11
Chronic myeloid leukemia	34	35.3 (12)	>0.05	-	-	18
Acute rejection of kidney graft	89	15.7 (14)	0.0056	0.44	0.24–0.80	91
Stable kidney graft function	196	26.0 (51)	>0.05	-	-	26
**Patients group vs Control II**						
Endometriosis	153	21.6 (33)	0.03	0.25	0.07–0.82	28
**Patients group vs Control III**						
Spontaneous abortion	149	26.2 (39)	>0.05	-	-	25

*healthy volunteers, including both groups listed below;

**healthy fertile women;

***women with two or more healthy-born children with the same partner and no history of abortion.

****statistical power was calculated post-hoc for alpha error level = 0.05.

*KIR2DS5* frequency in endometriosis was compared with that in healthy women who had delivered at least one healthy baby (Control II). Again, it was less frequent in the patients than in the controls (Table 1).

Women suffering from spontaneous abortion were compared with healthy women who had delivered at least two healthy children (Control III). No significant difference between these two groups was detected in frequencies of *KIR2DS5* gene (Table 1).

HLA-C C1 and C2 phenotype and genotype frequencies were significantly different from controls in ankylosing spondylitis (i.e. a decrease in C1 frequency) (Table 2). Moreover, C1C2 heterozygosity was less frequent in non-small-cell lung carcinoma patients (significant difference from the control, Table 2). However, both differences lost significance after correction.

**Table 2:** Frequencies of *HLA-C* C1 and C2 gene groups in different diseases.

HLA-C	Controls I (N = 680)	Controls II (N = 213)	AS* (N = 115)	RA* (N = 366)	E** (N = 153)	KGR* (N = 89)	KGNR (N = 196)	ALL (N = 21)	AML (N = 39)	CML (N = 33)	NSCLC (N = 269)
C1	83.7 (569)	82.2 (175)	73.9 (85)^a^	88.3 (323)	82.4 (126)	83.1 (74)	84.2 (165)	71.4 (15)	76.9 (30)	75.8 (25)	79.9 (215)
C2	67.2 (457)	64.8 (138)	74.8 (86)	69.1 (253)	60.1 (92)	75.3 (67)	66.3 (130)	76.2 (16)	59.0 (23)	63.6 (21)	61.0 (164)
C1C1	32.8 (223)	35.2 (75)	25.2 (29)	30.9 (113)	39.9 (61)	24.7 (22)	33.7 (66)	23.8 (5)	41.0 (16)	36.4 (12)	39.0 (105)
C2C2	16.3 (111)	17.8 (38)	26.1 (30)^b^	11.7 (43)	17.6 (27)	16.9 (15)	15.8 (31)	28.6 (6)	23.1 (9)	24.2 (8)	20.1 (54)
C1C2	50.9 (346)	46.9 (100)	48.7 (56)	57.4 (210)	42.5 (65)	58.4 (52)	50.5 (99)	47.6 (10)	35.9 (14)	39.4 (13)	40.9 (110)^c^

AS, ankylosing spondylitis; RA, rheumatoid arthritis; E, endometriosis; KGR, kidney graft rejectors; KGRN, kidney graft non-rejectors; ALL, acute lymphoblastic leukemia; AML, acute myeloid leukemia; CML, chronic myeloid leukemia; NSCLC, non-small cell lung carcinoma; Control I, healthy volunteers; Control II, healthy fertile women; *compared to Control I; **compared to Control II.

a *p* = 0.02 *OR* = 0.55 *95%CI* 0.35–0.88.

b *p* = 0.02 *OR* = 1.81 *95%CI* 1.14–2.89.

c *p* = 0.006 *OR* = 0.67 *95%CI* 0.50–0.89.

In addition, the analysis of combinations of the presence of *KIR2DS5* with the two HLA-C groups revealed several interesting results (see Table 3 for raw data and Table 4 for statistical analysis). In *KIR2DS5*-positive AS patients the frequencies of the *HLA-C* C1 phenotype were significantly lower than in Control I, and this was the only result in Table 4 which persisted significant after correction (p_c_ = 0.033). In AS, the C1C2 genotype was three times less frequent than in the controls. In contrast, the C2 phenotype frequency was decreased in endometriosis in comparison with Control II.

**Table 3:** Frequencies of *KIR2DS5* gene and C1 and C2 groups of *HLA-C* in several human diseases and healthy control individuals.

2DS5	HLA-C	Controls I (N = 680)	Controls II (N = 213)	AS* (N = 115)	RA* (N = 366)	E** (N = 153)	KGR* (N = 89)	KGNR* (N = 196)	NSCLC* (N = 269)
+	C1	25.1 (171)	25.5 (54)	10.4 (12)	20.8 (76)	18.3 (28)	13.5 (12)	21.4 (42)	21.6 (58)
+	C2	19.7 (134)	23.5 (50)	11.3 (13)	16.4 (60)	11.1 (17)	10.1 (9)	16.8 (33)	15.2 (41)
+	C1C1	9.8 (67)	8.5 (18)	5.2 (6)	7.9 (29)	10.5 (16)	5.6 (5)	9.2 (18)	10.8 (29)
+	C2C2	4.4(30)	6.6 (14)	6.1 (7)	3.6 (13)	3.3 (5)	2.2 (2)	4.6 (9)	4.5 (12)
+	C1C2	15.3 (104)	16.9 (36)	5.2 (6)	12.8 (47)	7.8 (12)	7.9 (7)	12.2 (24)	10.8 (29)
-	C1	58.4 (398)	56.8 (121)	63.5 (73)	67.5 (247)	64.1 (98)	69.7 (62)	62.8 (123)	58.4 (157)
-	C2	47.6 (323)	41.3 (88)	63.5 (73)	52.7 (193)	49.0 (75)	65.2 (58)	49.5 (97)	45.7 (123)
-	C1C1	22.9 (156)	26.8 (57)	20.0 (23)	23.0 (84)	29.4 (45)	19.1 (17)	24.5 (48)	28.3 (76)
-	C2C2	11.9 (81)	11.3 (24)	20.0 (23)	8.2 (30)	14.4 (22)	14.6 (13)	11.2 (22)	15.6 (42)
-	C1C2	35.5 (242)	30.1 (64)	43.5 (50)	44.5 (163)	34.6 (53)	50.6 (45)	38.3 (75)	30.1 (81)

AS, ankylosing spondylitis; RA, rheumatoid arthritis; E, endometriosis; KGR, kidney graft rejectors; KGRN, kidney graft non-rejectors; NSCLC, non-small cell lung carcinoma; Control I, healthy volunteers; Control II, healthy fertile women.

**Table 4:** Statistical analysis of the data from Table 3 (KIR2DS5, C1 and C2 frequencies).

Comparison	2DS5	HLA-C	*p*	*OR*	*95%CI*	Effect
AS vs Control I	+	C1	0.0003	0.35	0.19–0.65	↓
	+	C2	0.037	0.52	0.28–0.95	↓
	+	C1C2	0.003	0.31	0.13–0.71	↓
	-	C2	0.0017	1.92	1.28–2.89	↑
	-	C2C2	0.024	1.85	1.11–3.09	↑
RA vs Control I	-	C1	0.005	1.47	1.13–1.92	↑
	-	C1C2	0.005	1.45	1.12–1.88	↑
E vs Control II	+	C2	0.0025	0.41	0.22–0.74	↓
	+	C1C2	0.012	0.42	0.21–0.83	↓
KGR vs Control I	+	C1	0.017	0.47	0.25–0.89	↓
	+	C2	0.04	0.47	0.23–0.95	↓
	-	C1	0.0088	1.89	1.18–3.05	↑
	-	C2	0.0015	2.13	1.34–3.37	↑
	-	C1C2	0.0051	1.89	1.21–2.95	↑
KGR vs KGRN	-	C2	0.015	1.91	1.14–3.21	↑

AS, ankylosing spondylitis; RA, rheumatoid arthritis; E, endometriosis; KGR, kidney graft rejectors; KGRN, kidney graft non-rejectors; Control I, healthy volunteers; Control II, healthy fertile women; ↑, susceptibility; ↓, protection; *p*, probanility; *OR*, odds ratio; 95% *CI*, 95% confidence interval.

Similarly, in the absence of *KIR2DS5*, the C1 and C2 phenotypes were significantly associated with several clinical conditions. Thus C1 frequencies were increased in RA and kidney graft rejection, whereas those of C2 were increased in AS, and kidney rejection. C1C2 heterozygosity was increased in RA and kidney rejection.

No differences in the distributions of C1 and C2 phenotypes and genotypes combined with the presence or absence of *KIR2DS5* were observed in kidney graft nonrejectors (Tables 3 and 4) or patients with malignancies (ALL, AML, CML, and NSCLC; data not shown). The same was true for spontaneous abortion couples compared with Control III (data not shown).

## Discussion

Our results suggest that the presence of *KIR2DS5* gene may protect against some clinical conditions. In addition to psoriasis vulgaris described earlier [4], such protection was observed in endometriosis, ankylosing spondylitis, and acute rejection of kidney graft. In women with endometriosis, in contrast to healthy ones, ectopic endometrium is thought to be inefficiently eliminated by NK cells which exhibit insufficient cytotoxic activity in these patients [5], [6]. Therefore, a contribution of activating KIR2DS5 receptor to protection against endometriosis seems likely.

In contrast, an explanation of the apparently paradoxical protective effect of a killer cell-activating receptor in autoimmune disease (ankylosing spondylitis) and acute transplant rejection is more difficult. NK cells have been postulated to contribute to allograft tolerance induction [7], [8]. Therefore we may speculate that the presence of KIR2DS5 contributes to the activation of NK cells, leading to a more efficient induction of tolerance and inhibition of allograft rejection and autoimmunity. In contrast, lack of KIR2DS5 would weaken NK cell activation, resulting in insufficient induction of tolerance and in the promotion of acute kidney allograft rejection or autoaggression, for example in ankylosing spondylitis. It should be noted here, however, that in *HLA-B27*-positive Chinese patients with ankylosing spondylitis, *KIR2DS5* was associated rather with susceptibility than with protection compared with *HLA-B27*-positive controls [9], whereas it was not associated with this disease in *HLA*-unselected Chinese patients compared with unselected controls [10]. However, Chinese are genetically distant from Caucasians, as reflected, for example, by the difference in distribution of HLA-B27 subtypes (alleles) in these two populations [11], [12], and indeed, neither *KIR3DL1/KIR3DS1* [13] nor *KIR2DS5* [9] effects on AS in Chinese could be reproduced in some Caucasian populations [14], although they were observed in other Caucasians [15], [16]. In our study, we have not seen any association of KIR3DL1, putative receptor of HLA-B27 [17], [18], and only weak association of KIR3DS1, with ankylosing spondylitis (data not shown). This may speak against linkage disequilibrium (LD) of *KIR2DS5* with other *KIRs* as a main cause of its protective effect in AS.

One might argue that the apparent effect of *KIR2DS5* in other diseases might be caused by its LD with other *KIR2D* genes whose products bind HLA-C molecules, distinguishing between C1 and C2 epitopes [1], [2]. Indeed, kidney graft rejection seems to be associated in Poles with *KIR2DS4* full length gene (our unpublished data), which is in negative LD with *KIR2DS5* in many human populations including European Caucasians [19]. Therefore, the apparent protective effect of *KIR2DS5* described here for kidney rejection may result from this LD. On the other hand, only *KIR2DS5* gene was (negatively) associated with susceptibility to endometriosis, and *KIR2DS4* deletion variant was less frequent in peritoneal form of this disease (our unpublished data). This finding speaks against LD as an explanation for *KIR2DS5* protective effect in endometriosis because *KIR2DS4* and *KIR2DS5* are mutually exclusive in most haplotypes [19].

We also observed effects of *HLA-C* C1 and C2 genotypes in some diseases. We found an association with C2 homozygosity in ankylosing spondylitis, which seems to be concordant with results in Chinese, where an association of the HLA-Cw*02 allele (a member of the C2 group) with this disease was described [9]. We can not exclude LD of C2 with HLA-B27 as a main cause of this association, as most HLA-B*27 carriers in Caucasian populations have HLA-Cw*02 [20]. Another finding was that C1C2 heterozygosity seemed to protect from NSCLC. Although no data have been published on an association between NSCLC and *HLA-C* so far, we may speculate that the C1C2 genotype may allow for a wider spectrum of KIRs to contribute to control over cancer cells.

Additionally, we observed that in some diseases, the effect of C1 and C2 phenotypes or genotypes seemed to be detectable only in the absence of *KIR2DS5* gene but was visible only in its presence in other diseases. However, these results are easily explainable by the protective effect of *KIR2DS5* alone. For example, both the C1 and the C2 frequencies were reduced in AS patients positive for *KIR2DS5*, whereas both were increased in *KIR2DS5*-negative patients.

In summary, our results suggest protective effect of the presence of *KIR2DS5* gene in ankylosing spondylitis, endometriosis and acute kidney graft rejection, independent from *HLA-C*. Some *KIR2DS5*-independent associations of ankylosing spondylitis and NSCLC with *HLA-C* C1 and/or C2 groups are also reported here. To our knowledge, this is the first report on possible associations of non-small cell lung cancer with *KIR* and *HLA-C* genes.

## Materials and Methods

### Participants

Three hundred and sixty-six patients with rheumatoid arthritis (280 women, 64 men; mean age, 57.8±12.3, range 22–90) were recruited from the inpatient and outpatient population of the Clinic of Rheumatology and Internal Diseases, Autonomous Public Clinical Hospital No. 1, Pomeranian Medical University in Szczecin, Poland, and diagnosed according to the criteria of the American College of Rheumatology as described previously [21].

Two hundred and eighty patients (80 women and 200 men, mean age 62.06±9.3, range 35–87 years) with pathologically documented non-small-cell lung carcinoma (NSCLC) were qualified in the Department of Pulmonology and Lung Cancer, Wroclaw Medical University, and in the Clinic of Lung and Thorax Neoplasms, Centre of Oncology – Maria Skłodowska-Curie Institute, Warsaw. The diagnosis of lung cancer was based on positive histological or cytological examination (according to WHO criteria) [22]. A fibro-bronchoscopic examination was performed in all the patients, with harvesting of material for histological and cytological examination for establishing a diagnosis of NSCL. All participants underwent a sputum cytology survey as well. Advancement of the disease was determined according to the TNM staging system; thoracic computed tomography involving the upper abdomen was used for this purpose.

Two hundred and eighty-five adult cadaveric donor renal transplant recipients (123 women, 162 men; mean age, 43.43±11.44 years, range, 15–72) from the Department of Nephrology and Transplantation Medicine, Wrocław Medical University, who had undergone primary transplantation between 1989 and 2008 were included in the study; mean time of follow-up was 7 years. All patients received conventional immunosuppression: cyclosporine or tacrolimus in combination with azathioprine or mycofenolate mofetil and steroids. They were divided into two groups according to the occurrence of an acute rejection episode: 89 patients with an acute rejection episode and 196 patients without. Acute rejection was defined by an increase in serum creatinine level of at least 20% above the baseline measurements (not attributable to another cause) and confirmed by biopsy according to the Banff histopathological criteria [23]. Apart from three patients, all had a biopsy-confirmed acute rejection episode.

One hundred and fifteen patients (18 women, 83 men; age data for 101 patients were available: mean age, 36.7±14.5 years; range, 18–71) were diagnosed as definite ankylosing spondylitis in accordance with the modified New York classification criteria [24] at the Department of Rheumatology III, Silesian Hospital of Rheumatology and Rehabilitation, Ustroń, or at the Department and Clinic of Rheumatology and Internal Medicine, Wrocław Medical University. Ninety percent of the patients were HLA-B*27 positive by either serological or genetic typing (data not shown), in agreement with other populations [12].

Ninety-six patients treated with allogeneic hematopoietic stem cell transplant in the Department of Hematology and Bone Marrow Transplantation of the Silesian Medical University in Katowice because of leukemia (53 women, 43men; mean age, 33.3±10.9 years; including acute lymphoblastic leukemia, ALL, 22 patients, 11 women, 11 men; mean age, 27.4±9.7; acute myeloid leukemia, AML, 40 patients, 26 women, 14 men; mean age, 36.9±12.1; chronic myeloid leukemia, CML, 34 patients, 16 women, 18 men; mean age, 33.1 years) were described elsewhere [25].

Six hundred and ninety unrelated healthy volunteers (355 women, 335 men; mean age, 38.62±14.60, range, 19–56) were recruited in the years 2001–2008 by the Regional Center of Blood Transfusion, Wrocław, as well as by clinics of the Wrocław Medical University, the Medical University of Warsaw, and the Pomeranian Medical University, Szczecin (Control I, used for the diseases mentioned above).

One hundred and fifty-three women with endometriosis (mean age 33.7±8.0, range 20–58 years) diagnosed at the First and Second Departments of Obstetrics and Gynecology, Medical University of Warsaw, for pelvic pains, dysmenorrhea, and/or infertility were described in detail elsewhere [26]. Endometriosis had been confirmed and classified in these patients by both laparoscopic and histopathological examinations according to the revised American Fertility Society criteria [27]. Two hundred and thirteen healthy women who had given birth to at least one child were used as a control (Control II; this group included women from Control III described below and was included in Control I).

One hundred forty-nine couples (mean age of women: 32.44±4.21 years, range: 24–46) who had experienced spontaneous abortion (SA) were recruited for our study at the Polish Mother's Memorial Hospital - Research Institute, Łódź, the First Chair and Clinic of Obstetrics and Gynecology, Medical University of Warsaw, and the Institute of Human Genetics, Polish Academy of Sciences, Poznań. The criteria for patient classification and inclusion were presented elsewhere [28]. For a control (“Control III”), 117 healthy couples (mean age of women: 31.37±6.36, range: 23–68 years) with at least two healthy-born children and no history of abortion were included (Wrocław Medical University and Medical University of Warsaw).

### DNA isolation and *KIR2DS5* and *HLA-C* C1/C2 group typing

Genomic DNA was isolated from venous blood as described elsewhere [21], [29]. *KIR2DS5* was typed by either individual [29] or multiplex [30] polymerase chain reactions which, when compared in the same samples, gave concordant results. It should be noted here that our *KIR* typing is validated three times per year by the International KIR Exchange program organized by the Immunogenetics Center of the University of California at Los Angeles. *HLA-C* groups C1 and C2 were established as described by Frohn et al. [31].

### Ethics

This project was accepted by the Bioethics Committee of the Medical University of Warsaw, the Bioethics Committee of the Wrocław Medical University, the Bioethics Committee of the Pomeranian Medical University in Szczecin, the Bioethics Committee of the Polish Mother's Memorial Hospital - Research Institute in Łódź, the Bioethics Committee of the Silesian Medical University in Katowice, and the Bioethics Committee of the Medical University of Poznań. Signed informed consent was given by all participants. Ludwik Hirszfeld Institute of Immunology and Experimental Therapy has no Bioethic Committee for research on human materials, and our projects are evaluated by the Bioethics Committee of the Wrocław Medical University. The same applies to the Silesian Hospital of Rheumatology and Rehabilitation which needs approval from the Bioethics Committee of the Silesian Medical University in Katowice. Approval from the Bioethics Committee of the Institute of Oncology in Gliwice was not required, as no samples originated from that institution.

### Statistical methods

Differences between controls, patients, and patient subgroups were estimated using the two-tailed Fisher's exact test and GraphPad InStat 3 software. A *p* value<0.05 was considered significant. The Bonferroni correction of *p*-values (if<0.05) was calculated for the number of comparisons (11 for Table 1, 45 for Table 2, and 70 for Table 3). Statistical power was calculated post-hoc for alpha error level = 0.05.
